# Can frozen-section analysis of ureteric margins at the time of radical cystectomy predict upper tract recurrence?

**DOI:** 10.1080/2090598X.2020.1751923

**Published:** 2020-04-17

**Authors:** Karim Soliman, Diaa-Eldin Taha, Omar M. Aboumarzouk, Islam Osama Koraiem, Ahmed A. Shokeir

**Affiliations:** a Department of Urology, Urology and Nephrology Center, Mansoura University, Mansoura, Egypt; b Department of Urology, Faculty of Medicine, Kafrelsheikh University, Kafrelsheikh, Egypt; c Glasgow Urological Research Unit, Department of Urology, Queen Elizabeth University Hospital, Glasgow, UK; d School of Medicine, Dentistry and Nursing, University of Glasgow, Glasgow, UK; e Department of Urology, Damanhour International Medical Institute, Beheira, Egypt

**Keywords:** Bladder cancer, frozen-section analysis, upper urinary tract recurrence, radical cystectomy

## Abstract

**Objective:**

To summarise the currently available literature and analyse available results of the outcome of intraoperative frozen-section analysis (FSA) on upper urinary tract recurrence (UUTR) after radical cystectomy (RC).

**Materials and methods:**

A systematic review of the literature was performed according to the Cochrane Reviews guidelines and in accordance with the Preferred Reporting Items for Systematic Reviews and Meta-Analyses (PRISMA) checklist. Articles discussing ureteric FSA with RC were identified.

**Results:**

The literature search yielded 21 studies, on which the present analysis was done. The studies were published between 1997 and 2019. There were 10 010 patients with an age range between 51 and 95 years. Involvement of the ureteric margins was noted in 2–9% at RC. The sensitivity and specificity of FSA were ~75% and 99%, respectively. Adverse pathology on FSA and on permanent section, prostatic urothelial carcinoma involving the stroma but not prostatic duct, and ureteric involvement on permanent section were all more likely to develop UUTR. Neither evidence of ureteric involvement nor ureteric margin status on permanent section were significant predictors of overall survival.

**Conclusion:**

Routine FSA is mandatory for a tumour-free uretero–enteric anastomosis and is predictive of UUTR. To lower the UUTR, FSA is not necessary if the ureters are resected at the level where they cross the common iliac vessels. FSA is indicated whenever the surgeon encounters findings suspicious of malignancy, e.g. ureteric obstruction, periureteric fibrosis, diffuse carcinoma *in situ*, induration or frank tumour infiltration of the distal ureter is discovered unexpectedly during surgery, and prostatic urethral involvement.

**Abbreviations:**

CIS: carcinoma *in situ*; FSA: frozen-section analysis; HR: hazard ratio; PRISMA: Preferred Reporting Items for Systematic Reviews and Meta-Analyses; RC: radical cystectomy; (UT)UC: (upper tract) urothelial carcinoma; UUT(R): upper urinary tract (recurrence)

## Introduction

Bladder cancer represents the fifth most common malignancy in the Western world, with an incidence of 80 470 in 2019 and a mortality rate of 17 670 per year in the USA alone [1,2].

The incidence of upper urinary tract recurrence (UUTR) after radical cystectomy (RC) is reportedly 2.4–6.6%, and is associated with multiple clinical and pathological risk factors including tumour multifocality, pathological stage, presence of carcinoma *in situ* (CIS), and ureteric and urethral involvement [3–6].

To identify ureteric margin status, and thus ureteric involvement, intraoperative frozen-section analysis (FSA) during RC and then serial sectioning of the distal ureter is performed. The validity of this approach in improving UUTR outcomes is controversial, with several publications questioning the accuracy of FSA and the feasibility of achieving uninvolved ureteric margins by sequential ureteric sectioning in light of the relative rarity of UUTR after RC [7].

To this end, we aimed to conduct a systematic review of the literature to evaluate the accuracy of FSA to detect malignant ureteric margins at the time of RC and to determine the impact of final margin status obtained by a sequential sectioning strategy on the risk of UUTR.

## Materials and methods

### Search strategy and study selection

The systematic review was performed according to Cochrane Review Guidelines and in accordance with the Preferred Reporting Items for Systematic Reviews and Meta-Analyses (PRISMA) checklist [8].

The search strategy was conducted to find relevant studies from the Medical Literature Analysis and Retrieval System Online (MEDLINE; 1966–2019), the Excerpta Medica dataBASE (EMBASE; 1980–2019), Google Scholar, and different urological journals. The search was conducted in March 2019.

The search terms used included: ‘bladder cancer’, ‘upper tract recurrence’, ‘radical cystectomy ‘, and ‘intraoperative ureter frozen section’.

Mesh phrases included:

(‘Radical cystectomy’[Mesh]) AND “Upper tract recurrence “[Mesh]) ((“Upper tract recurrence “[Mesh]) AND ‘Radical cystectomy’[Mesh]) AND ‘Intraoperative ureter frozen section’[Mesh]), (((‘Radical cystectomy’[Mesh]) AND ‘Upper tract recurrence’[Mesh]) AND ‘Intraoperative ureter frozen section, Radical cystectomy’[Mesh]) AND ‘upper tract recurrence’[Mesh])

#### Inclusion criteria

1. All studies reporting on FSA of the distal ureter during RC and the impact on UUTR.

2. Studies published in the English language over the period 1980–2019.

#### Exclusion criteria

1. Animal studies and case reports.

2. Studies on RC and diversion that did not look at the ureteric margins or UUTR.

The references of the retrieved papers were evaluated for potential inclusion. Authors of the included studies were contacted wherever data were not available or not clear. Two reviewers (K.S. and D.E.T.) identified all studies that adhered to the inclusion criteria for full review. Each reviewer independently selected studies for inclusion. Disagreement between the extracting authors was resolved by consensus or referred to a third author (A.A.S.).

### Data extraction and analysis

The objectives were to evaluate the impact of intraoperative FSA on UUTR after RC. The following variables were extracted from each study: number of patients, study origin and date, population demographics, tumour characteristics, UUTR, overall and disease-specific survival. The data from each study were grouped into an analysis on an intention-to-treat basis, to allow a numerical representation of the results.

## Results

**Figure 1.**
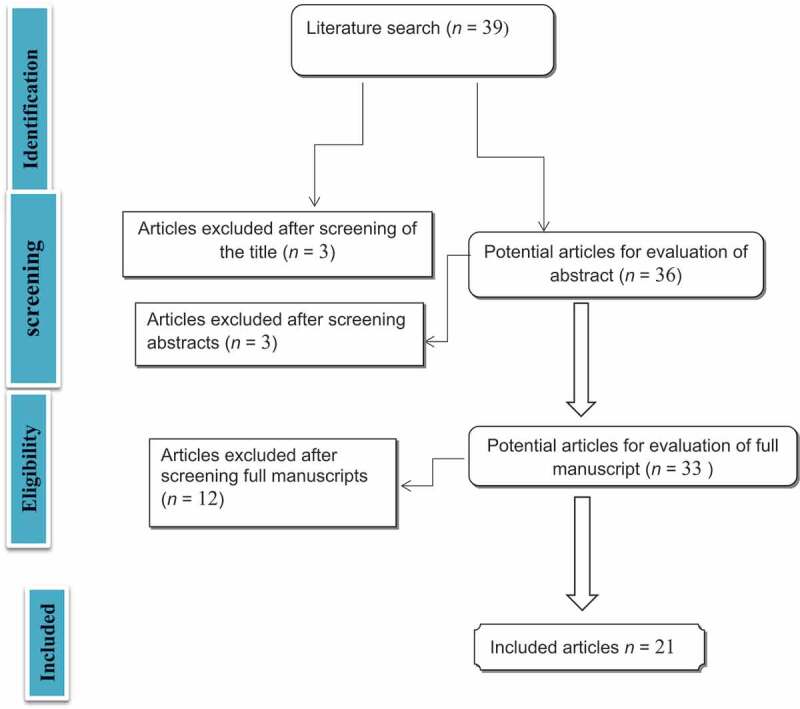
Flowchart of article selection.

The literature search yielded 39 studies, of which 18 were excluded because of irrelevance of data (Figure 1), as the titles and abstracts of the studies did not give sufficient data on UUTR after RC. All included studies were retrospective studies, with no randomisation or control groups.

All studies reported on the variables indicated in the data extraction section and are listed in Table 1 [1,3,4,6,7,9–23].

**Table 1.** The 21 included studies on ureteric FSA at RC.

							Stage, n (%)								
Authors	Study type	Patients, *n*	Follow-up, months	+ve FSA, *n* (%)	+ve Final pathology, *n/N* (%)	CIS, *n/N* (%)	<T2	T3	T4	LVI, *n*	+ve LNI, *n*	UUTR, *n/N* (%)	NAC, *n*	ACT, *n*	Ureteric recurrence, *n/N* (%)
Moschini et al., 2016 [1]	Retro.	1447	95	368 (25)	190/368 (52)	122/368 (33)	144	133	90	97	138	35	13	55	26
Volkmer et al., 2009 [19]	Retro.	1420	58	-		10	13	-	-		-	2			25
Kim et al., 2014 [20]	Retro.	402	50	54/645 ureters (8.4)		93	243	159		136	86	9	41	113	9
Huguet-Pérez et al., 2001 [3]	Retro.	568	60	-		17	11	15			-	20			20
Sved et al., 2004 [6]	Retro.	235	42	-		2						1			5
Lee et al., 2006 [18]	Retro.	115	30	12		3						22			
Silver et al., 1997 [7]	Retro.	401	23	31		25	10	20			-	-			3
Balaji et al., 1999 [4]	Retro.	529	17	0		1	5	11			1	9			16
Raj et al., 2006 [9]	Retro.	1330	3.8 years	9% of ureters (13% of patients)		767 (58)									
Satkunasivam et al., 2016 [17]	Retro.	2047	12.4 (1.9–10.1) years			567 (27.7)	1297 (63.3)	541 26.4)	119 (10.2)				132 (6.5)	427 (20.9)	No
Gakis et al., 2011 [13]	Retro.	218	24	17/425 (4.0)		71	110	73	35	62	56	5/218 (2.3)	NA	NA	NA
Tollefson et al., 2010 [22]	Abstract	1134	21	146 (12.9)	27 (2.4)							50 (4.4)			
Schumacher et al., 2006 [15]	Retro.	805										31/805 (3.9)			2/805 (0.3)
Loeser et al., 2014 [26]	Abstract	243	26	1/117 patients (0.85) in Group I (without CIS) and 21/59 patients (35.6) in Group II (with concomitant CIS)		59						In Group II, 2 patients (1.1)			
Johnson et al., 1989 [11]	Retro.	217	24	8/217						0	0		0	0	0
Schoenberg et al., 1996 [10]	Retro.	101	67	6 (41)	3/14	8%				0	0	1	0	0	0
Sharma et al., 1970 [14]	Retro.	205				17/205									1
Cooper et al., 1973 [23]	Retro.	48		8 (18)								3/13			
Culp et al., 1967 [21]						13/231 (6)									
Touma et al., 2010 [12]	Retro.	301		36/602	23/602	2.8%									
Osman et al., 2007 [16]	Prosp.	193		16 (8.3)		15%									

ACT: adjuvant chemotherapy; LNI, lymph node invasion; LVI: lymphovascular invasion; NA: not available; NAC: neoadjuvant chemotherapy; Prosp.: prospective; Retro.: retrospective.

### Characteristics of the included studies

The studies were published between 1997 and 2019. There were 10 010 patients (84% male vs 16% female) with an age range between 51 and 95 years. The follow-up period ranged between 17 and 148 months.

### Incidence of ureteric involvement at RC

The involvement of the ureteric margins at the time of RC was reported in the range of 2% to 9% at the time of RC [7,9–14,24].

An association in the incidence TCC and CIS in the resected ureteric margins was found in 4.8%. In only 1.2% of them was it found at the level of iliac crossing and more proximally resected ureteric segments [15]. There was a lot of evidence for an association between bladder CIS and the incidence of ureteric involvement, at 30% (107 patients) vs 9% (22) for patients with and without bladder CIS, respectively [25,26].

The incidence of ureteric dysplasia was 6.9% in patients who underwent RC with preoperative radiotherapy compared to only 3.2% in patients who underwent RC without preoperative irradiation [11].

Bilateral presentation of the disease, which was either synchronous or metachronous, was reported in 13% of all upper tract urothelial carcinomas (UTUCs). The results from a study from Taiwan found that there were no significant differences in contralateral recurrence-free survival between various bladder tumour stages and the grade of the tumour. Bladder cancer presence, either previously metachronous or synchronous, did not predict contralateral recurrences (*P* = 0.14) [27].

### *Diagnostic characteristics and utility of FSA* (Figure 2 [15])

FSA positivity of the ureteric margins for malignancy was found in the presence of CIS or invasive UC, while negative margins were found in patients that had reactive atypia or low-grade dysplasia [9].

**Figure 2.**
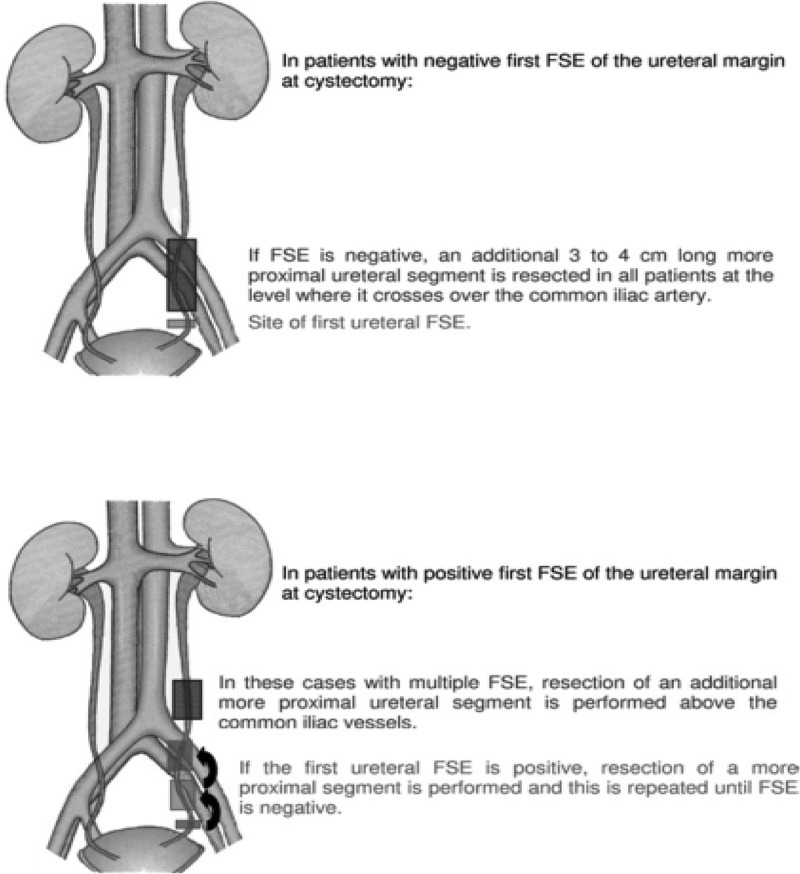
Template of how to perform a frozen-section examination [15].

The sensitivity and specificity of FSA was ~75% and 99%, respectively [9,12,15,16]. In comparison with final histopathological analysis as the ‘gold standard’, the false-positive and false-negative rates for FSA were 2% and 6%, respectively [7,9,10,17,26].

A positive FSA, tumour multifocality [13], male gender, CIS, and concomitant urethral malignancy [6,9,12,16] were independent predictors of true distal ureteric malignancy. Mucosal or ductal prostatic UC (odds ratio 1.78, 95% CI 1.11–2.86), but not stromal invasion, was associated with adverse ureteric margin pathology at FSA in males after adjusting for age, pathological grade and stage [11,13,15,17].

Compared to the corresponding permanent pathological examinations, the intraoperative ureteric FSA detected CIS in 17% [7] or in 75% [15].

Positive ureteric margins were noted in 9% of patients on permanent pathological section and in only 10% of those in whom intraoperative frozen sections were sent [9].

### Number of frozen sections taken

Successful intraoperative conversion to benign pathology, after two or more FSAs occurred in 77 ureters (66.4%) [17]. Meanwhile, tumour-free margins were achieved after a single subsequent resection in only seven of 17 ureters (41.1%) and in the other 10 ureters (58.9%) tumour-free margins were not achieved at FSA despite subsequent re-sectioning of up to three times [median (range) 1 (1–3); *P* = 0.48] [13].

The ‘serial step sectioning’ strategy was successful in converting only 28% of ureters to a negative FSA margin. Of these, 38 were ‘converted’ from a positive margin on FSA to an uninvolved ureteric anastomotic margin. Overall, 48 of 124 ureters (39%) with an initial positive margin on FSA were converted to a negative margin on permanent section (4% of 1217 ureters in which FSA was performed) [9].

### Are abnormal FSA ureteric margins associated with UUTR?

UUTR occurred in 2–7% of patients undergoing RC [3,4,9,28]. Some other studies noted an incidence of 2.3% [13,18,26,29]. Moreover, the risk is greater, up to 17%, in patients with CIS at the ureteric margin on FSA [15,19]. UT carcinoma is rare and the commonest form is UC, other histopathology, e.g. squamous cell carcinoma and adenocarcinoma are very rare [30].

After adjustment for age, stage, grade, and presence of CIS, in patients with adverse pathology on FSA and on permanent section and prostatic UC involving the stroma [hazard ratio (HR) 3.3, 95% CI 1.09–9.97; *P =* 0.034), but not prostatic ducts (HR 2.54, 95% CI 0.74–8.78; *P =* 0.14) [17], and ureteric involvement on permanent section (HR 1.8, 95% CI 1.1–3.1; *P = *0.048), were more likely to develop UUTR than those without ureteric involvement [9].

There was 100% concordance with the side of UTUC recurrence and the side of initial adverse pathology, despite the successful conversion to benign intraoperative margins in 90% of cases (and benign permanent sections of the proximal ureter) [17].

The renal pelvis was frequently the first site of recurrence (68%) followed by the uretero–enteric anastomosis (16%) and the ureters (16%) [9].

### Does the resection of abnormal margins reduce the risk of uretero–enteric anastomotic recurrence?

The incidence of an invasive tumour recurrence at the uretero–enteric anastomosis after RC is ~1% [9,11,31,32].

Ureteric margin CIS was most frequently found in patients with multifocal tumours, and those with high-stage and high-grade disease [14].

No patient, with either dysplasia or CIS developed a ureteric malignancy in the area of uretero–enteric anastomosis after a median follow-up of 6 years [10,11]. Conversely, of CIS diagnosed on the first ureteric FSA, one patient had recurrence at the site of uretero–enteric anastomosis. In one patient ureteric FSA was normal, whereas in the other patient CIS was diagnosed [15,19,20].

On the other hand, recurrent transurethral resection of bladder tumour (TURBT) indicates recurrent tumour, unhealthy urothelium and high-grade tumour, which are all risk factors for positive FSA and increase risk of recurrence. From different articles there was a statistically significant variance in recurrence in patients with a history of multiple urothelial recurrences or with multifocal tumours when compared to patients who has a solitary lesion at pre-RC TURBT [19,27]

### Value of FSA on survival

Neither evidence of ureteric involvement (HR 0.9; 95% CI, 0.7–1.1) nor ureteric margin status (HR 1.0, 95% CI 0.7–1.3) on permanent section were significant predictors of overall survival [9,17,33].

Sanderson et al. [29] reported a survival rate of 73% in patients with low tumour stages (pTa–pT1) at a median of 3.4 years, meanwhile in advanced stages (pT3) only 8% were alive at a median of 1.2 years. As the risk of ureteric malignancy at the time of RC is highest in the distal part of the ureter [15] and neoadjuvant chemotherapy has the potential of tumour down-staging, patients with evidence of tumour involvement of the distal ureter in the preoperative staging may derive benefit from neoadjuvant chemotherapy to reduce the risk of malignant ureteric margins at RC [34]. FSA of the distal ureters at RC is however unlikely to be positive unless the bladder cancer stage is ≥T2 [33].

When comparing the stages of N0 vs N+ (including all patients with lymph node involvement by the urothelial lesion) there is a large difference in incidence in both groups. Patients with nodal involvement have potential short-term follow-up related to their high mortality rate, so we cannot conclude the precise effect of nodal stage on UUTR [35].

### Cost, cost-effectiveness, and alternatives to FSA

Each ureteric FSA generates a charge somewhere in the vicinity of 400 USD and may increase the overall pathology charges by 25% or 50% if one or two FSAs are evaluated per each ureter [9,10]. The cost to identify one patient with CIS or solid UC of the ureter on FSA was 6471 USD [12].

## Discussion

The investigators hypothesised that FSA of the ureteric margins at the time of RC would support complete removal of the tumour, which would in turn improve survival of patients with bladder cancer. Culp et al. [21] first discussed this matter after discovering that 38 of 231 patients (17%) had unexpected ureteric epithelial abnormalities at RC, so they suggested that unappreciated CIS could result in recurrence in the remaining ureter and renal pelvis, although their series was small and a lot of the patients had advanced disease at follow-up.

The initial stimulus for FSA was to ensure a cancer-free anastomosis, which supposedly would transform into reduced UUTR rates and longer cancer-free survival. Yet, controversy remains concerning the value of ureteric margin FSA in achieving such goals due to the rarity of UUTR [15]. FSA is mostly indicated whenever the surgeon encounters findings suspicious of malignancy, e.g. ureteric obstruction, periureteric fibrosis, diffuse CIS, induration or frank tumour infiltration of the distal ureter that is discovered during the surgery, and involvement of the prostatic urethra [10,11]. FSA of the ureters is not necessary if the ureters are resected at the level where they cross the common iliac vessels [15].

An UUTR was defined as any documented radiographic, cystoscopic, or pathologically confirmed recurrence in the kidneys, ureters, or urinary diversion. Documentation of a mass within the ureter, renal pelvis or ureteric wall thickening with enhancement on intravenous contrast administration was considered radiographic evidence of urothelial recurrence. While, ‘ureteric wall thickening without enhancement’, ‘ureteric streaking’, ‘streaky changes around the ureter’, ‘periureteric stranding’, ‘fullness/infiltration around the ureter’, ‘ureteric dilation’, ‘renal masses not involving the renal pelvis’, or ‘worsening hydronephrosis’, were considered insufficient evidence of urothelial recurrence [9].

An association with TCC and CIS was found in 4.8% in the wide range of the distal ureter and in 1.2% at the level of iliac cross and more proximally resected ureteric segments [15]. Evidence of bladder CIS is associated with higher incidence of ureteric involvement (30% vs 9%, for patients with and without bladder CIS, respectively) [25,26].

The positivity of ureteric margins for malignancy is evident in the presence of CIS or invasive UC, while, negative when histological analysis demonstrated reactive atypia or low-grade dysplasia [9].

It has been suggested that a more proximal ureteric segment can be excised to assure a tumour-free uretero–enteric anastomosis, evident by the highest incidence of urothelial malignancies in the distal ureter [31]. Therefore, resection of the ureters at the level where they cross over the common iliac arteries should increase the probability of a tumour-free uretero–enteric anastomosis [15].

The sensitivity and specificity of FSA was ~75% and 99%, respectively [9,12,15,16]. In comparison with final histopathological analysis as the ‘gold standard’, the false-positive and false-negative rates for frozen section were 2% and 6%, respectively [7,9,10,17,26]. Compared to the corresponding permanent pathological examinations, the intraoperative ureteric FSA can only detect CIS in 17% [7] or in 75% [15].

The incidence of an invasive tumour recurrence at the uretero–enteric anastomosis after RC was ~1% [9,11,31,32]. Ureteric margin CIS was most frequently found in patients with multifocal tumours and those with high-stage and high-grade disease [14].

Tumour multifocality [13], male gender, CIS, and concomitant urethral malignancy [6,9,12,16] were independent predictors of true distal ureteric malignancy. Mucosal or ductal prostatic UC, but not stromal invasion, was associated with adverse ureteric margin pathology at FSA [11,13,15,17]. On the other hand, recurrent TURBTs indicate recurrent tumour, unhealthy urothelium and high-grade tumour, which all are risk factors for positive FSA and increase the risk of recurrence. From different articles there was a statistically significant variance in recurrence in patients with a history of multiple urothelial recurrences or with multifocal tumours when compared with patients who had a solitary lesion at pre-RC TURBT [19].

The presence of a JJ stent could be a risk factor for UUTR if drainage of the UUT is performed. Kiss et al. [36] reported UUTR in 1005 patients who had concomitant bladder tumours, 114 (11%) of whom had drainage, including in 53 (46%) with JJ stenting and 61 (54%) with percutaneous nephrostomy. They reported UUTR in 31 patients (3%) at a median of 17 months after RC, including seven of the 53 (13%) in the JJ-stent group, 0% in the nephrostomy group, and 24 of 891 (3%) in the no drainage group. Multivariate regression analysis revealed a higher risk of UUTR if patients underwent JJ stenting (HR 4.54, 95% CI 1.43–14.38; *P* = 0.01) [36].

The number of frozen sections that should be taken during RC is variable. One biopsy may be sufficient, but two or more may be needed in order to reach benign pathology that at the same time does not affect ureteric length [13,17].

The ‘serial step sectioning’ strategy involves serial cutting of the distal ureter, beyond the last 0.5 cm of the vesico-ureteric junction, until reaching a negative FSA. Sequential sectioning of the ureter aims to eliminate all cancer tissue, yet, this objective remains incomplete if tumour tissue remains at the primary surgical site or node metastases are present. It was successful in converting up to 28% of ureters to a negative FSA margin [9]. A negative FSA margin in the majority of patients was not reached during sequential ureteric resection due to technical considerations in anastomosing ureters that are too short, the limited length of residual ureters, and minimising potential complications of uretero–enteric anastomotic strictures [7,10].

The FSA should also be evaluated from a cost perspective. Each ureteric FSA generates a charge somewhere in the vicinity of 400 USD [9,10,12]. Any costs can be justified when one considers the cost implications of further treatments or surgery if recurrences occur.

An UUTR occurred in 2–7% of patients undergoing RC [3,4,9,13,26,28,29]. Moreover, the risk increases in patients with CIS at the ureteric margin on FSA [15]. Concomitant UUT tumour with bladder cancer occurs in 0.7–2.6% [37].

After adjustment for age, stage, grade, and presence of CIS, patients with adverse pathology on FSA and on permanent section and prostatic UC involving the stroma, but not prostatic ducts [17], and ureteric involvement on permanent section, were more likely to develop UUTR than those without ureteric involvement [9]. Both a positive initial and final margin status was associated with UUTR. Those patients with a positive initial margin were 5.3-times more likely to experience an UUTR than those with a negative initial margin. Of those with a positive initial margin, patients with a positive final margin were 60% more likely to experience an UUTR than those with a negative final margin [22].

The renal pelvis was frequently the first site of recurrence (68%), followed by the uretero–enteric anastomosis (16%), and the ureters (16%) [9]. The definitive treatment for such evident recurrence is either nephroureterectomy or ureteric resection and the creation of an ileal ureter, if a solitary renal unit is the actual situation [17,23].

Neither evidence of ureteric involvement (HR 0.9, 95% CI 0.7–1.1) nor ureteric margin status (HR 1.0, 95% CI 0.7–1.3) on permanent section were significant predictors of overall survival [9,17]. However, patients with CIS at the ureteric margin that was not diagnosed by FSA subsequently might have pelvic recurrence, distant metastases, and radiographic evidence of UUTR [10,15].

As the risk of ureteric malignancy at the time of RC is highest in the distal part of the ureter [15] and neoadjuvant chemotherapy has the potential of tumour down-staging, patients with evidence of tumour involvement of the distal ureter at preoperative staging may derive benefit from neoadjuvant chemotherapy to reduce the risk of malignant ureteric margins at RC [34].

## Conclusion

Routine FSA should not be ignored, in order to achieve tumour-free uretero–enteric anastomosis and to decrease the incidence of UUTR. It is not required if the ureters are resected at the level above where they cross the common iliac vessels. It is indicated whenever the surgeon encounters findings suspicious of malignancy, e.g. ureteric obstruction, periureteric fibrosis, diffuse CIS, induration or frank tumour infiltration of the distal ureter is discovered unpredictably during surgery, and prostatic urethral involvement. Tumour multifocality, male gender, CIS, and concomitant urethral malignancy were independent predictors of true distal ureteric malignancy. Mucosal or ductal prostatic UC, but not stromal invasion, were associated with adverse ureteric margin pathology at FSA.
